# Slimmer Geminals For Accurate F12 Electronic Structure Models

**DOI:** 10.1021/acs.jctc.5c00971

**Published:** 2025-09-10

**Authors:** Samuel R. Powell, Kshitijkumar A. Surjuse, Bimal Gaudel, Edward F. Valeev

**Affiliations:** Department of Chemistry, 1757 Virginia Tech, Blacksburg, Virginia 24060, United States

## Abstract

The Slater-type F12 geminal length scales originally tuned for the second-order Mo̷ller-Plesset F12 method are too large for higher-order F12 methods formulated using the SP (diagonal fixed-coefficient spin-adapted) F12 ansatz. The new geminal parameters reported herein reduce the basis set incompleteness errors (BSIEs) of absolute coupled-cluster singles and doubles F12 correlation energies by a significant—and increase with the cardinal number of the basis—margin. The effect of geminal reoptimization is especially pronounced for the cc-pV*X*Z-F12 basis sets (specifically designed for use with F12 methods) relative to their conventional aug-cc-pV*X*Z counterparts. The BSIEs of relative energies are less affected, but substantial reductions can be obtained, especially for atomization energies and ionization potentials with the cc-pV*X*Z-F12 basis sets. The new geminal parameters are therefore recommended for all applications of high-order F12 methods, such as coupled-cluster F12 methods and transcorrelated F12 methods.

## Graphical abstract

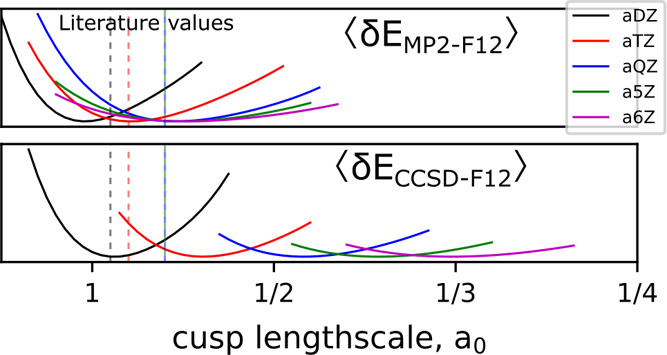

## 1 Introduction

The notoriously slow basis set convergence of the electronic energies and other properties evaluated with correlated electronic structure models arises primarily because of the lack of many-electron cusps in the conventional Fock space basis of products of single-particle states. To reduce the basis set incompleteness error (BSIE), one can extrapolate the property of interest to the complete basis set limit using one of the families of systematically designed basis sets, namely the correlation-consistent bases^1–3^ or the atomic natural orbital (ANO) bases.^4–7^ Although some basis set extrapolation formulas can be rationalized,^8^ many formulas in use are largely ad hoc recipes^9–12^ whose reliability is only as good as the amount of available benchmark data. The bulk of basis set extrapolation testing has concentrated on light elements and the correlation-consistent basis set family, though studies of the robustness of basis set extrapolation for energies obtained with ANO bases and for heavy elements (where ANOs and basis sets containing effective core potentials (ECPs) are the dominant choices) are ongoing.^13–25^

The first-principles solution to the basis set problem is to use the so-called explicitly correlated models, which contain Fock space basis functions that model the many-electron cusps by including explicit dependence on the interelectronic distances. Following the pioneering work of Hylleraas^26^ and Slater^27,28^ many such models^29–31^ have been developed targeting the high-precision simulation of small systems as well as applicability to larger systems.

There are several groups of explicitly correlated approaches in use today:

- High-precision methods: these involve unfactorizable many-particle integrals with most or all particles at once that are evaluated analytically, for example, Hylleraas-CI,^32^ explicitly correlated Gaussian geminals,^33^ and the free iterative complement interaction (ICI) approach of Nakatsuji.^34^
- Configuration-space Jastrow factor-based methods: these are methods involving unfactorizable many-particle integrals of most or all particles at once that are evaluated stochastically or avoided by serving as a trial wave function; examples include single- and multiconfiguration Slater-Jastrow wave functions in variational and diffusion quantum Monte Carlo methods.^35^
- Configuration-space transcorrelation: this involves unfactorizable integrals with at most 3 particles that are evaluated analytically.^36,37^

- R12/F12 methods: these involve unfactorizable integrals with up to 4 particles that are evaluated analytically (up to 2-particle integrals) or by the resolution-of-the-identity (3- and 4-particle integrals).^29–31,38,39^

By only requiring the exact evaluation of (nonstandard) 2-particle integrals, the F12 methods are most practical among all explicitly correlated methods, available routinely in several packages and with demonstrated applications to hundreds and thousands of atoms.^40–43^

Other key advantages of modern F12 methods over most other explicitly correlated methods are that (a) no problem-specific nonlinear optimization of the parameters of the explicitly correlated terms is required and (b) the spin-dependence of the cusp conditions^44^ can be satisfied rigorously, unlike the purely configuration-space methods.^45^ Following Ten-no,^46^ the standard formulation of F12 methods uses a single Slater-type geminal (STG)

$$f_{\beta }\left(r_{12}\right)≡-\frac{\exp\left(-\beta r_{12}\right)}{\beta }$$ 1

to correlate every pair of electrons in the molecule. The recommended value of *inverse length scale* β was preoptimized for valence-only^3^ and all-electron^47^ correlated computations with the most common orbital basis sets, but it is kept fixed in the course of the computation, and no other adjustable parameters (linear or nonlinear) are associated with the explicitly correlated terms. The use of system-independent preoptimized parameters should be contrasted with the other types of explicitly correlated methods, almost all of which involve optimization of adjustable nonlinear parameters of the explicitly correlated terms, which impacts their robustness. For example, to improve robustness, Szenes et al. recently argued^48^ that simpler system-independent correlators are more likely to be useful in the context of transcorrelated methods, which is fully in the spirit of the F12 methods.

The lack of adjustable parameters, however, may be limiting the accuracy of the F12 methods relative to the other explicitly correlated analogs. All available evidence^49–51^ suggests that the STG in eq 1 is near optimal when used to correlate valence electron pairs. Another way to improve the accuracy would be to go back to the older “orbital-invariant” ansatz,^52^ which correlated each electron pair using an adjustable linear combination of *O* ^2^ (where *O* is the number of occupied orbitals) explicitly correlated terms generated from a single fixed geminal; unfortunately, this ansatz suffered from geminal superposition errors^53^ and poor conditioning of its optimization.^54^ Using multiple geminals^55^ is another possibility but would suffer from the same issues as the orbital-invariant approach.

Fortunately, it turns out that there is still room for improvement of the standard F12 technology using single fixed STGs. In the course of stretching the F12 technology to be usable with large (6Z and 7Z) correlation-consistent basis sets, we noticed that the optimal geminal length scale for the modern F12 formalism differs relatively strongly between low-order models like MP2-F12 and infinite-order models like CC-F12 (albeit with approximate inclusion of the F12 terms). Unfortunately, the recommended length-scale parameters available in the literature were all determined at the MP2-F12 level of theory. Namely, Peterson and co-workers^3^ determined the optimal geminal parameters by maximizing the magnitude of the MP2-F12 correlation energy of a collection of atoms and molecules spanning the second and third periods of the Periodic Table; they provided recommended geminal length scales for the augmented correlation-consistent basis sets^1,2,56–58^ aug-cc-pV*X*Z (abbreviated as a*X*Z herein) for *X* = D,T,Q,5 and the F12-specialized correlation-consistent basis sets^3^ cc-pV*X*Z-F12 (abbreviated as *X*Z-F12 herein) for *X* = D,T,Q (see Table 1). Later, Hill et al. performed a somewhat more robust optimization, again at the MP2-F12 level of theory, which resulted only in minor changes to the recommended exponents.^59^ The latter approach was also used to provide a recommended exponent for the 5Z-F12 basis.^60^ Although Hill et al. noticed that the optimal geminal exponents differed significantly between the MP2-F12 and CCSD-F12 methods, they attributed the difference to an artifact of the particular CCSD-F12 approximation (namely, the F12b approach)^61^ used in their work, in part because the CCSD-F12b energies obtained with the larger optimal exponents they found for F12b overshot their CBS limit estimate, which was obtained from CCSD-F12b computations with a large custom uncontracted basis. As additional evidence, they referred to an earlier study by Tew et al., which concluded that the optimal geminal exponents agree well between MP2-F12 and another CCSD-F12 approximation (the CCSD­(F12) method).^62^ The study of Tew et al. used the older F12 formalism for explicitly correlated MP2 and CCSD based on the nondiagonal orbital-invariant ansatz^52^ rather than the more modern F12 formalism based on the SP ansatz of Ten-no^63^ (i.e., the diagonal F12 ansatz with amplitudes fixed by the exact spin-dependent cusp conditions, denoted with the “(fix)”^64^ suffix by the Molpro team). Note that Knizia et al. had also observed the difference in the geminal exponent dependence of the SP-ansatz-based F12b and non-SP-ansatz (F12) coupled-cluster counterparts.^65^ Hill et al. did not associate the observed MP2 vs CC geminal length-scale differences with the use of the diagonal ansatz, although some earlier evidence in a sufficiently different setting^66^ indicated that the diagonal F12 ansatz has a stronger dependence on the F12 geminal length scale than the nondiagonal counterpart, suggesting the diagonal and nondiagonal F12 results should be compared with caution.

**Table 1:** Optimal β Values Recommended in This Work

OBS family	source	training method	D	T	Q	5	6	7
aXZ	ref 3	MP2-F12	1.1	1.2	1.4	1.4	–	–
	ref 59	MP2-F12	1.0	1.2	1.4	1.5	–	–
	**this work**	**MP2-F12**	**0.96**	**1.21**	**1.46**	**1.49**	**1.51**	–
	**this work**	CCSD-F12	**1.12**	**1.61**	**2.16**	**2.57**	**2.98**	**3.85**
XZ-F12	ref 3	MP2-F12	0.9	1.0	1.1	–	–	–
	ref 59	MP2-F12	0.9	1.0	1.0	1.2^a^	–	–
	**this work**	**MP2-F12**	**0.84**	**0.93**	**0.92**	**1.02**	**–**	**–**
	**this work**	CCSD-F12	**1.06**	**1.52**	**1.95**	**2.31**	**–**	**–**

a Obtained in ref 60 using the optimization method of ref 59.

In our own testing, we also observed a difference in the optimal geminal exponents between the MP2-F12 and CCSD-F12 methods using a perturbative approximation to the CCSD-F12 (namely, the CCSD(2) $\overset{¯}{F12}$ method).^67,68^ Thus, it became clear that it is necessary to reoptimize the geminal length scales for high-order F12 methods, such as the CC-F12 method as well as the transcorrelated F12 method.^69,70^ This study reports our initial findings and the set of recommended geminal length scales for application with MP2-F12 and CC-F12 methods using the a*X*Z (*X* = D···6) and *X*Z-F12 (*X* = D···5) basis sets. Additionally, we provide a recommendation for the CC-F12 method on the a7Z basis. In Section 2 we describe the technical details of computations. Section 3 describes our protocol for optimizing β and the analysis of the optimal values and their effect on the basis set incompleteness of absolute and relative correlation energies. In Section 4, we summarize our findings.

## 2 Technical Details

All the results reported herein were produced with a development version of the Massively Parallel Quantum Chemistry software package.^71^ All correlation energies were obtained with only valence electrons correlated (frozen-core approximation). Both MP2 and CCSD-F12 methods utilized the SP ansatz.^63^ The explicitly correlated CCSD energies were produced using the perturbative CCSD(2) $\overset{¯}{F12}$ approximation^67,68,72,73^ to the full CCSD-F12 method; for simplicity, we use CCSD-F12 to denote the former. The F12 two-electron basis was generated from a single STG^46^ (eq 1), without the usual approximation as a linear combination of Gaussian Geminals.^49,51,74^ Integrals of the STG and related integrals can be reduced to the core one-dimensional integral,^46,75^

$$G_{m}\left(T,U\right)=\int_{0}^{1}dt\;t^{2m}\exp\left(-Tt^{2}+U\left(1-t^{-2}\right)\right),m\leq -1$$ 2

which in the Libint Gaussian AO integral engine are computed using 15th-order Chebyshev interpolation for 0 ≤ *T* ≤ 2^10^, 10^–7^ ≤ *U* ≤ 10^3^. For larger *T,* the upward recursion relation^46^ is used. Until this work, combinations of *T* and *U* outside these ranges have not been encountered. However, the combination of low exponents present in high-*X* OBS and higher β than previously considered requires the evaluation of eq 2 with *U* ≥ 10^3^ and small *T*. To support the evaluation of integrals of the STG and related integrals for an extended range of Gaussian AO exponents and geminal parameters, Libint version 2.11 ^76^ introduced a new approach. Namely, outside of the ranges covered by the interpolation and upward recursion, the STG is represented as a linear combination of Gaussian geminals using the approach developed in refs 77 and 78 and used in ref 79. with exponents and coefficients provided by trapezoidal quadrature of its integral representation

$$-\frac{\exp\left(-\beta r\right)}{\beta }=-\frac{2}{\sqrt{\pi }}\int_{-\infty }^{\infty }ds\;\exp\left(s-\beta^{2}\exp\left(2s\right)-r^{2}\exp\left(-2s\right)/4\right)$$ 3

on interval *s* ∈ [log­(ϵ)/2 – 1, log­(*Tr*_lo_^–2^)/2] discretized evenly with step size *h* = (0.2 – 0.5log _10_(ϵ))^−1^; *T* = 26 is sufficient to ensure {relative, absolute} precision of ϵ = 10^–12^ for *r* between *r*_lo_ = 10^–5^ and {β^–1^,*∞*}, respectively.

Molecular orbitals were expanded in Dunning’s aug-cc-pV*X*Z^1,2,56,57,80–85^ orbital basis sets (OBSs), denoted a*X*Z, as well as the cc-pV*X*Z-F12 basis sets of Peterson and co-workers,^3,60,86^ denoted as *X*Z-F12. Robust density fitting in the aug-cc-pV*X*Z-RI^87,88^ density fitting basis set was used to approximate the 2-electron integrals throughout all computations. 3- and 4-electron integrals in the special F12 intermediates were approximated using the CABS+ approach^89^ and approximation C.^90^ The aug-cc-pV*X*Z/OptRI^91^ and cc-pV*X*Z-F12/OptRI^92^ auxiliary basis sets (ABS) were used to approximate the F12 intermediates in computations with the a*X*Z and *X*Z-F12 OBS, respectively. a*X*Z/OptRI, *X*Z-F12/OptRI, and a*X*Z-RI basis sets are only available for *X* ≤ 5, *X* ≤ Q, *X* ≤ 6, respectively; hence, for computations with higher *X,* we used the largest respective basis that is available, e.g., a6Z and a7Z OBSs were matched by the a5Z/OptRI ABS. The only exceptions were computations with 5Z-F12 OBS; the a5Z/OptRI basis set was used instead of QZ-F12/OptRI. Significant errors were observed when extrapolating energies with the a7Z results computed with the a6Z-RI basis set. Thus, we used an automatically generated density fitting basis set using the MADF approach described in ref 93.

All Gaussian AO basis sets not already included in the Libint library^76^ were obtained from the Basis Set Exchange,^94,95^ except the a7Z basis for hydrogen, which was provided by John F. Stanton’s research group.

The CBS CCSD valence correlation energies and their contributions to the atomization energies were obtained by the *X* ^–3^ extrapolation^10^ of the a6Z and a7Z energies. The corresponding CCSD contributions to the reaction energies and ionization potentials (IPs) used *X* ^–3^ extrapolation from the aQZ and a5Z energies. The “Silver” benchmark CCSD values from Řezáč et al.’s original paper were used as the CCSD reference binding energies for the S66 benchmark.^96^

## 3 Results

### 3.1 Geminal Length-Scale Optimization

The optimal inverse length scale β_opt_ for the given combination of OBS and F12 method was determined by minimizing the following objective function:

$${\mathrm{E̅}}_{\mathrm{F12}}\left(\beta \right)=\left\langle \frac{E_{\mathrm{F12}}^{S}\left(\beta \right)}{E_{\mathrm{F12}}^{S}\left(\beta_{\mathrm{opt}}^{S}\right)}\right\rangle$$ 4

where *E*_F12_^*S*^(β) is the F12 contribution to the energy of system *S*, β_opt_^*S*^ is the value of β that minimizes *E*_F12_^*S*^(β), and ⟨···⟩ denotes the averaging over *S*. The purpose of the denominator in eq 4 is to renormalize each fit according to the energy of each system to avoid giving excessive weight to the systems with large correlation energies, thereby balancing the correlation physics across the periods and groups of the Periodic Table.

To reduce the cost of optimization, *E*_F12_^*S*^(β) for each system was approximated by a quartic polynomial obtained by least-squares fitting *E*_F12_^*S*^(β) evaluated on an equidistant grid of β values (use of the sixth-order polynomial changed the optimal exponents by 0.01 or so). For each basis/method combination, the grid was a set of points $\beta_{i}≡i/20,i\in Z$ selected such that at least 3 grid points were included on each side of β_opt_^*S*^ for every *S* in the training set. The training set of systems {*S*} included the lowest singlet states of dimers A_2_ and hydrides AH_*x*_, with A including elements from groups 13 to 17 in the second and third periods of the Periodic Table, as well as the Ne and Ar atoms. Namely, the benchmark set consists of B_2_, BH_3_, C_2_, CH_4_, N_2_, NH_3_, O_2_, H_2_O, F_2_, HF, Ne, Al_2_, AlH_3_, Si_2_, SiH_4_, P_2_, PH_3_, S_2_, H_2_S, Cl_2_, HCl, and Ar. All computations were performed at the equilibrium CCSD­(T)/aTZ geometries obtained from CCCBDB.^97^ Since the a7Z basis is not available for the third-period elements, only the molecules composed of second-period elements were used for the geminal optimization with that basis. Additionally, treating CH_4_ with the a7Z basis proved too challenging due to noisy DF errors with the recently designed MADF basis, so it was omitted from the optimization set in this case.

The resulting optimal geminal parameters β_opt_ as well as the recommended values from refs. 3,59,60 are listed in Table 1.

There is excellent agreement between our MP2-F12 β_opt_ and those obtained previously by Peterson and co-workers, despite the differences in the training sets and technical details; most deviations are smaller than 0.1. The largest deviation of 0.18 is observed for the cc-pV5Z-F12 basis; as we will see shortly, tolerance of small errors in β_opt_ rapidly increases with *X* due to the rapid decrease in the curvature of *E̅*_F12_(β) near the minimum.

The most notable insight from Table 1 is the rapid increase in the gap between CCSD-F12 and MP2-F12 β_opt_; while for the double-ζ basis sets, the gap is modest (≈0.2) and exceeds 1 for quintuple-zeta basis sets and apparently continues to grow thereafter. It is noteworthy that the β_opt_ obtained by Hill et al. using CCSD-F12b with the a5Z basis, 2.4 *a*_0_^–1^,^59^ is in good agreement with our β_opt_ value, 2.57 *a*_0_^–1^, despite the substantial differences between the iterative CCSD-F12b approximation and perturbative CCSD(2) $\overset{¯}{F12}$ approximation to exact CCSD-F12. Plots of *E̅*_F12_(β) in Figure 1 illustrate the consequences of using MP2-F12-optimized exponents for large OBS: although the dependence on β weakens with *X,* and thus, high-*X* F12 energies are more tolerant of small errors in β_opt_, the CCSD-MP2 gap β_opt_ is large and grows with *X*; hence, using MP2-F12-optimized β_opt_ will result in suboptimal CCSD-F12 energies for *X* ≥ 3.

**Figure 1:**
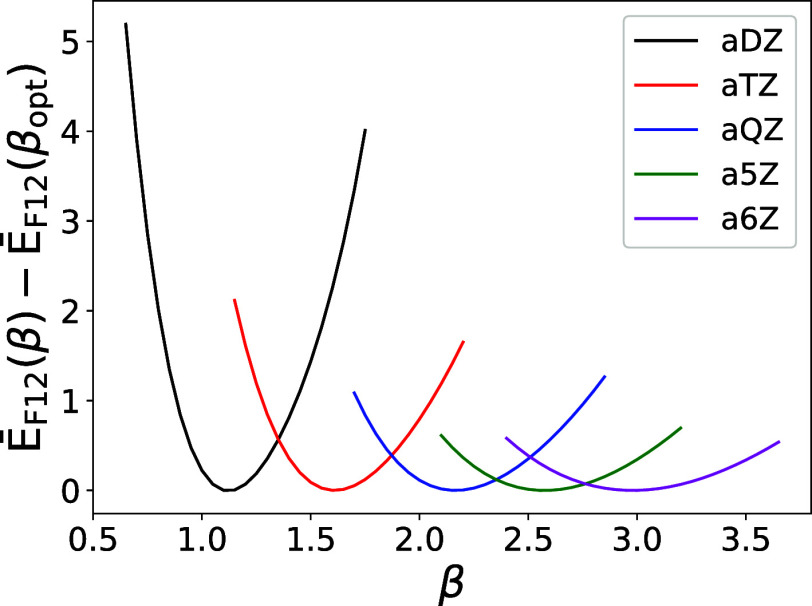
Levelized CCSD-F12 *E̅*_F12_(β) for the a*X*Z OBS family. Optimal β increases with cardinal number *X*, whereas the curvature of *E̅*_F12_(β) decreases with *X*.

Table 2 illustrates quantitatively the dependence of CCSD-F12 BSIE on the geminal length scale. The fifth column illustrates the reduction of the CCSD-F12 BSIE by switching from the original geminal length scales of Peterson et al. to our per-basis optimal CCSD-F12 parameters listed in Table 1; the BSIEs are reduced by more than 60% for quadruple-ζ basis sets and by more than a factor of 2 for quintuple-zeta basis sets. The use of CCSD-F12 rather than MP2-F12 optimized geminal parameters for yet larger OBS results in progressively larger BSIE reductions, by a factor of 4 for a7Z. Clearly, the use of CCSD-F12-optimized geminal parameters for CCSD-F12 and other infinite-order F12 methods should be preferred.

**Table 2:** Average Basis Set Incompleteness Errors of CCSD-F12 Correlation Energies (m*E*_h_) Obtained with the Recommended Geminal Parameters of Ref 3,60 (β_ref_; β_ref_ = 1.4 Was Used for a6Z and a7Z MP2-F12), Our Per-Basis Recommended CCSD-F12 Parameters from Table 1 (β_opt_) and Their System-Optimized Counterparts (β_opt_^*S*^)­^a^

OBS	⟨δ(β_ref_)⟩	⟨δ(β_opt_)⟩	⟨δ(β_opt_^*S*^)⟩	⟨δ(β_ref_)/δ(β_opt_)⟩	⟨δ(β_ref_)/δ(β_opt_^*S*^)⟩	⟨δ(β_opt_)/δ(β_opt_^*S*^)⟩
aDZ	17.42	17.30	16.02	1.005	1.095	1.087
aTZ	7.76	6.11	5.91	1.244	1.287	1.032
aQZ	3.48	2.10	2.04	1.646	1.691	1.030
a5Z	1.71	0.85	0.83	2.187	2.235	1.024
a6Z	1.11	0.45	0.44	2.513	2.584	1.029
a7Z	0.36	0.08	0.08	4.855	4.899	1.008
DZ-F12	13.05	12.04	11.29	1.082	1.153	1.060
TZ-F12	5.07	3.86	3.75	1.414	1.453	1.028
QZ-F12	1.95	1.20	1.17	1.913	1.958	1.023
5Z-F12	1.03	0.51	0.50	2.592	2.651	1.020

a δ­(β) ≡ *E*_corr_^CCSD‑F12^(β) – *E*_corr_^CBS^.

Note that the optimal geminal parameters vary substantially across the period and group, even for valence electrons. Figure 2 illustrate the distribution of β_opt_^*S*^ across the training set {*S*}. Several trends are noticeable. First, β_opt_^*S*^ varies more strongly across the second period and less strongly across the third period. Second, variation across the period weakens with increasing *X*. Third, β_opt_^*S*^ for the third period of elements is nearly universally smaller than that for the second period. Despite the noticeable variation of β_opt_^*S*^ across the period and group of the Periodic Table, system-specific optimization of β results in a relatively small benefit compared to the use of per-basis β_opt_, as illustrated by the last column of Table 1. Namely, the BSIE is only reduced by ≈3% by system-specific optimization. Considering that the use of basis-tuned rather than system-tuned geminal parameters greatly simplifies the practical use of F12 methods (by, e.g., preserving their size consistency), in our opinion, system-specific geminal optimization in the context of F12 methods is not worthwhile.

**Figure 2:**
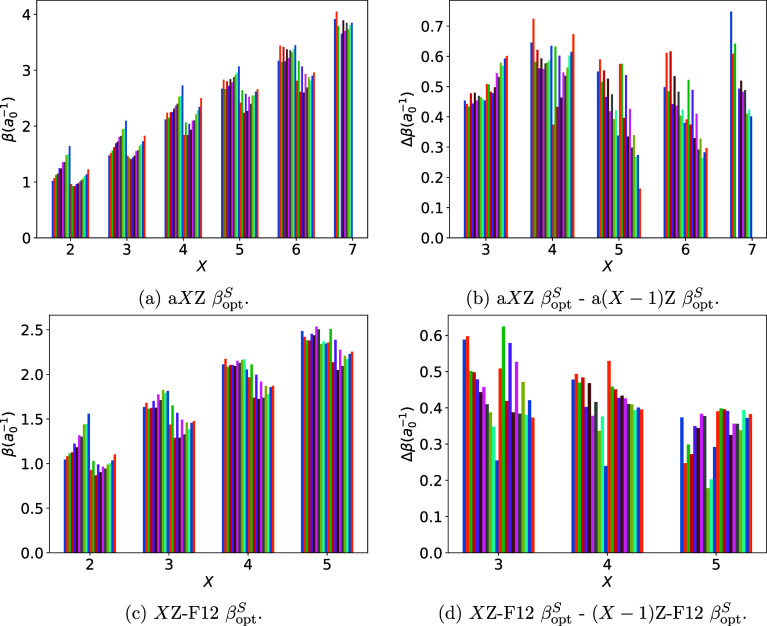
Distribution of CCSD-F12-optimized geminal parameters β_opt_^*S*^ for the (a) a*X*Z and (c) *X*Z-F12 basis sets and change in optimal parameter for the (b) a*X*Z and (d) *X*Z-F12 basis sets across the training set. The bars represent each system in the training set with the same order of systems as that listed in Section 3.1.

Variation with the period and group is observed not only for the position of the minimum of *E*_F12_^*S*^(β) (i.e., β_opt_^*S*^) but also its curvature, as illustrated by Figure 3. It is somewhat unexpected that for heavier elements, the F12 energy is more strongly dependent on β.

**Figure 3:**
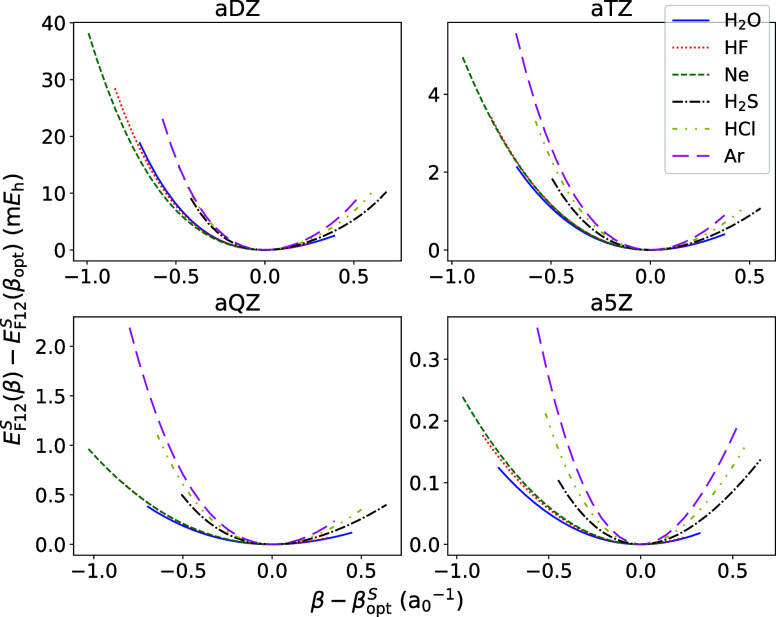
*E*_F12_^*S*^(β) – *E*_F12_^*S*^(β_opt_^*S*^) for several representative valence-isoelectronic systems with second- and third-period elements. Note the relatively weak variation of the curvature across the period, but substantial curvature increases upon transition from the second to third periods.

Figure 2 illustrate that β_opt_^*S*^ varies relatively regularly with *X*, although the distribution changes shape and the noticeable differences between dimers and hydrides become pronounced for large *X*.

The strong increase in β_opt_ upon transition from MP2-F12 to CCSD-F12 must correlate with the wider basis set error of the Coulomb hole of the MP1 wave function compared to its CCSD counterpart. Our findings agree with other observations regarding the differences between MP2 and infinite-order methods. For example, it is known that the basis set errors of MP2 are generally larger than those of higher-order methods like CCSD (e.g., see the relevant discussion in ref 67). Also note the substantial differences in the optimal unoccupied orbital basis for MP2 vs CCSD studied in ref 98.

### 3.2 Effect of Geminal Optimization on BSIEs of Relative Energies

Does the observed reduction in the BSIEs of *absolute* CCSD-F12 energies translate into reduced BSIEs of relative energies? To answer this question, we report the CCSD correlation BSIEs for a variety of relative energies obtained with β_ref_ and β_opt_ in Table 3. These include (1) a set of 15 reaction energies (see Table 9 in ref 62) involving small molecules with second- and third-period elements, (2) a set of 5 binding energies of weakly bound pairs of molecules selected from the S66 benchmark set (namely water dimer, ethyne dimer, methane dimer, water–methane pair, and ethyne-water pair),^96^ (3) IPs of homonuclear second-period diatomics (B_2_, C_2_, N_2_, O_2_, F_2_, at their experimentally derived equilibrium geometries^97^), and (4) atomization energies of several molecules from the HEAT data set (N_2_, H_2_O, HF, F_2_, OH, CH, NH_3_, at geometries revised by John F. Stanton’s research group and listed in Supporting Information).

**Table 3:** Statistical Analysis of the BSIEs of CCSD Correlation Energy Contributions to Various Relative Energies^a^

	aDZ		aTZ		aQZ		a5Z	
	β_ref_	β_opt_	β_ref_	β_opt_	β_ref_	β_opt_	β_ref_	β_opt_
	reaction energies							
⟨δ⟩	0.666	0.657	0.359	0.489	–0.167	–0.078	–0.064	–0.003
⟨|δ|⟩	1.915	1.920	0.958	1.343	0.624	0.692	0.404	0.421
σ	2.570	2.594	1.474	1.940	0.791	0.997	0.535	0.617
	noncovalent interaction energies							
⟨δ⟩	–0.478	–0.477	–0.353	–0.394	–0.202	–0.214		
⟨|δ|⟩	0.478	0.477	0.353	0.394	0.202	0.214		
σ	0.109	0.111	0.097	0.076	0.051	0.054		
	ionization potentials							
⟨δ⟩	95.6	95.3	24.8	24.1	5.9	6.2	3.1	2.4
⟨|δ|⟩	95.6	95.3	24.8	24.1	5.9	6.2	3.1	2.4
σ	59.6	58.6	20.4	18.9	5.1	4.4	1.6	1.5
	atomization energies							
⟨δ⟩	4.535	4.381	1.962	1.608	–0.271	–0.319	0.027	–0.132
⟨|δ|⟩	6.154	6.052	2.066	1.864	0.433	0.399	0.140	0.135
σ	5.567	5.551	1.609	1.498	0.523	0.453	0.182	0.129

	DZ-F12		TZ-F12		QZ-F12		5Z-F12	
	β_ref_	β_opt_	β_ref_	β_opt_	β_ref_	β_opt_	β_ref_	β_opt_
	reaction energies							
⟨δ⟩	0.508	0.493	0.186	0.258	0.096	0.098		
⟨|δ|⟩	1.415	1.591	0.594	1.049	0.467	0.566		
σ	1.723	1.832	0.845	1.472	0.692	0.853		
	noncovalent interaction energies							
⟨δ⟩	–0.118	–0.192	–0.167	–0.159	–0.063	–0.067		
⟨|δ|⟩	0.118	0.192	0.167	0.159	0.063	0.067		
σ	0.033	0.083	0.052	0.075	0.027	0.038		
	ionization potentials							
⟨δ⟩	76.1	68.9	23.0	19.0	8.7	5.6	4.1	2.0
⟨|δ|⟩	76.1	68.9	23.0	19.0	8.7	5.6	4.1	2.0
σ	44.8	38.5	14.6	13.2	4.8	4.3	1.6	1.7
	atomization energies							
⟨δ⟩	7.621	5.938	2.409	1.516	0.688	0.170	0.259	–0.079
⟨|δ|⟩	7.621	5.938	2.409	1.520	0.688	0.213	0.259	0.095
σ	3.347	2.996	1.268	1.000	0.378	0.173	0.143	0.151

a ⟨δ⟩, ⟨|δ|⟩, and σ denote the mean signed, mean unsigned, and standard deviation of BSIE, respectively, δ ≡ *E* – *E*_CBS_. IP BSIEs are in meV, and the rest of the values are kJ/mol.

As expected, relative CCSD-F12 correlation energies are less affected by the optimization of β than absolute energies. The BSIEs of reaction energies, in fact, generally appear to be marginally worse with β_opt_ than with β_ref_. The BSIEs of noncovalent interaction energies are only marginally affected by the changes in β. Ionization potentials and atomization energies are the only properties where significant improvement is observed for some basis sets. The most significant differences are observed for the *X*Z-F12 basis sets, but for the largest a*X*Z basis sets, some improvement is also observed. For example, even for the {T,Q}­Z-F12 OBS, the BSIEs of CCSD-F12 correlation atomization energies are reduced from {2.41, 0.69} to {1.52, 0.21} kJ/mol, i.e., by {37, 61} %. As expected, CCSD-F12 computations for the largest basis sets benefit most from the use of β_opt_.

The original motivation for this work was to reduce the BSIEs of coupled-cluster energies in accurate thermochemical benchmarks, such as HEAT,^99,100^ by replacing the high-end extrapolation of CCSD­(T) energies with extrapolation-free explicitly correlated CCSD­(T) energies. Optimization of the geminal parameters with the largest a6Z basis sets allows us, for the first time, to probe whether the F12 methods are competitive with extrapolation in this regime. Figure 4 illustrates the CCSD correlation contribution to atomization energies obtained by extrapolation and with CCSD-F12. Indeed, it appears that the F12 energies should be preferred to extrapolation, as the basis set convergence of the former appears more systematic and rapid than that of the latter. The optimization of β does not appear to have a significant effect on convergence, which is in agreement with the nearly identical performance of β_ref_ and β_opt_ a*X*Z CCSD-F12 for atomization energies in Table 3. According to the data in Table 3, we expect the convergence of *X*Z-F12 atomization energies to be substantially improved by the use of β_opt_. In any case, the BSIEs of the F12 energies seem to be significantly reduced relative to that of the extrapolated counterparts; the average unsigned (*X* = 6) – (*X* = 5) difference for the F12 β_opt_ energies is 0.077 kJ/mol, whereas the corresponding value for the a­{*X* – 1, *X*}­Z *X* ^–3^-extrapolated energies is 0.244 kJ/mol.

**Figure 4:**
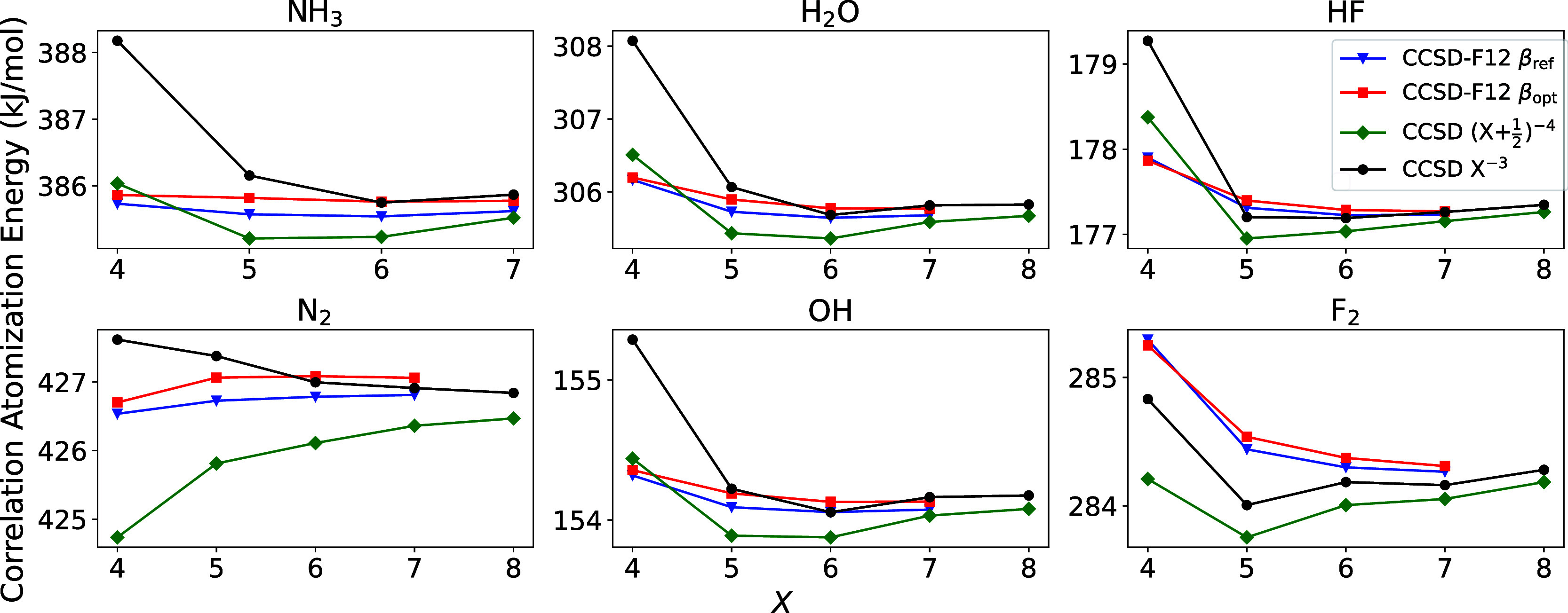
Basis set convergence of a*X*Z CCSD-F12 and a­{*X* – 1, *X*}­Z extrapolated CCSD correlation contributions to atomization energies of several small molecules in the HEAT benchmark set.^99^ β_ref_ and β_opt_ denote the use of geminal parameters from ref 3 (extended to use β_ref_ = 1.4 for a6Z OBS) and Table 1, respectively.

Although relative CCSD-F12 correlation energies are not as significantly affected by the reoptimization of β as are the absolute counterparts, in some important regimes the use of CCSD-F12-optimized β yield major reduction of BSIE. Thus, the use of the new CCSD-F12-optimized β values is recommended for F12 variants of all accurate (i.e., infinite-order) model chemistries, such as the CC-F12 methods.

## 4 Conclusions

We have reported updated recommendations for the geminal length-scale parameter for F12 calculations, showing that the previous values optimized using the MP2-F12 method^3,59^ are suboptimal for higher-order F12 methods formulated using the SP (diagonal fixed-coefficient spin-adapted) F12 ansatz. The new geminal parameters are shown to reduce the BSIEs of absolute valence CCSD-F12 correlation energies by a significant (and increasing with the cardinal number of the basis) margin. The effect of geminal reoptimization is especially pronounced for the cc-pV*X*Z-F12 basis sets (specifically designed for use with F12 methods) relative to their conventional aug-cc-pV*X*Z counterparts. The BSIEs of relative energies are less affected, but substantial reductions can be obtained, especially for atomization energies and ionization potentials with the cc-pV*X*Z-F12 basis sets. The new geminal parameters are therefore recommended for all applications of coupled-cluster F12 methods.

It remains to be seen how strongly the optimal geminal parameters depend on the form in which F12 terms are included and on the level of the correlation treatment. In limited testing, only negligible differences were found in the geminal exponents optimized with CCSD-F12 and CCSDT-F12 methods. Slightly larger differences were observed between the optimal exponents of traditional incorporation of F12 terms into the cluster operator of CC-F12 and their a priori introduction via the F12-style transcorrelation.^69,70,101^ A more thorough investigation of these effects is underway and will be reported elsewhere.

## Supplementary Material



## Notes

Molecular geometries, select raw computational data (PDF)
