# Supplementary material for: Slimmer Geminals For Accurate F12 Electronic Structure Models
Source: J Chem Theory Comput. 2025 Sep 10;21(18):8833–42. doi: 10.1021/acs.jctc.5c00971 (PMC12461919; doi:10.1021/acs.jctc.5c00971)
Supplement: Supplementary file 1 [file ct5c00971_si_001.pdf]

Slimmer Geminals For Accurate F12 Electronic Structure Models.

Samuel R. Powell, Kshitijkumar A. Surjuse, Bimal Gaudel, and Edward F. Valeev*

Department of Chemistry, Virginia Tech, Blacksburg, Virginia, 24060

E-mail: efv@vt.edu

1 Molecular Geometries

Below are molecular geometries for the 7 molecules included in the atomization set. These are HEAT geometries (in atomic units) revised by John F. Stanton's research group.

```
**** CH ****
2

C -0.00000000     0.00000000     0.16372625
H  0.00000000     0.00000000    -1.94946043
**** F2 ****
2

F -0.00000000     0.00000000     1.33445279
F  0.00000000     0.00000000    -1.33445279
**** H2O ****
```

```
3

H  0.00000000    -1.43108710     0.98391550
O  0.00000000    -0.00000000    -0.12399124
H  0.00000000     1.43108710     0.98391550
**** HF ****
2

H -0.00000000     0.00000000     1.64546855
F  0.00000000     0.00000000    -0.08728862
**** N2 ****
2

N -0.00000000     0.00000000     1.03701651
N  0.00000000     0.00000000    -1.03701651
**** NH3 ****
4

N   -0.12753001     0.00000000     0.0000000
H    0.59064885    -0.88517861    -1.5331743
H    0.59064885     1.77035723    -0.0000000
H    0.59064885    -0.88517861     1.5331743
**** OH ****
2

O -0.00000000     0.00000000     0.10862251
H  0.00000000     0.00000000    -1.72391805
```

****

2 Select Raw Data

Table S1: Reaction energies computed with the aXZ basis sets using Peterson's and our β_{opt} and Q5 extrapolated value

	aDZ		aTZ		aQZ		a5Z		CBS
Rxn.	β_{ref}	β_{opt}	β_{ref}	β_{opt}	β_{ref}	β_{opt}	β_{ref}	β_{opt}	
1	-26.4240	-26.4244	-27.8605	-27.3154	-28.4504	-28.0865	-28.0820	-27.9490	-27.7120
2	1.0466	0.9980	3.0359	2.7390	3.4086	3.3403	3.2652	3.2313	2.9916
3	60.0314	59.9334	57.6471	56.4789	57.8310	57.1236	57.7789	57.5223	58.6009
4	-51.4155	-51.4784	-48.1167	-48.4238	-48.5093	-48.6576	-47.6951	-47.7375	-46.9516
5	17.9118	17.8795	18.8580	19.7368	17.7844	18.0040	17.7863	17.9568	17.7619
6	5.0420	4.9948	5.5417	5.4296	5.4849	5.4645	5.6128	5.6664	5.8271
7	-21.1914	-21.1773	-23.6594	-23.3149	-24.0917	-23.8679	-23.7066	-23.5907	-23.3152
8	20.9325	20.9482	19.0364	19.2070	18.0401	18.0877	18.0809	18.1434	17.8679
9	-6.9226	-6.9081	-8.2664	-8.1840	-8.7051	-8.5336	-8.3421	-8.2612	-8.2027
10	11.2333	11.2367	10.6169	10.5831	10.5833	10.5517	10.6161	10.6306	10.6710
11	-5.1461	-5.1598	-6.3676	-6.5982	-6.2110	-6.1491	-6.0316	-5.9587	-5.8508
12	6.3431	6.2513	7.3043	6.9409	7.7895	7.7302	7.8220	7.8406	8.2621
13	92.9299	92.9608	90.3733	91.0558	87.3872	87.4771	86.9468	87.0484	86.0385
14	13.3612	13.5036	16.2501	17.4525	15.0792	15.9671	14.7482	15.0328	13.6140
15	3.0707	3.1065	1.8050	2.3636	0.8853	1.1887	1.0572	1.1947	1.2109

Table S2: Reaction energies computed with the XZ-F12 basis sets using Peterson's and our β_{opt} and Q5 extrapolated values

Rxn.	DZ-F12		TZ-F12		QZ-F12		CBS
	β_{ref}	β_{opt}	β_{ref}	β_{opt}	β_{ref}	β_{opt}	
1	-24.7962	-25.4801	-27.5027	-27.3256	-27.7851	-27.7570	-27.7120
2	3.4573	2.6088	3.4228	3.0806	3.2217	3.1492	2.9916
3	59.6444	58.6599	57.3227	55.7603	57.4069	57.0134	58.6009
4	-48.0735	-49.4117	-47.5485	-48.3344	-47.5588	-47.9977	-46.9516
5	14.8953	15.5299	17.6453	18.5343	18.2845	18.4280	17.7619
6	7.0656	6.7892	5.6538	5.4537	5.6744	5.7019	5.8271
7	-20.3853	-20.3840	-23.1572	-22.9782	-23.4778	-23.4180	-23.3152
8	19.4133	20.2251	18.7185	19.1717	18.3042	18.4085	17.8679
9	-5.8433	-6.0238	-7.7611	-7.6200	-8.2271	-8.1608	-8.2027
10	10.4460	10.8235	10.6023	10.5892	10.5362	10.5695	10.6710
11	-4.6016	-4.8258	-5.7521	-5.7389	-5.9201	-5.9028	-5.8508
12	8.3220	7.3500	7.4953	7.0031	7.8964	7.8111	8.2621
13	85.9182	88.5086	87.7679	89.1863	87.5434	87.7553	86.0385
14	11.1488	11.3700	15.5399	16.1721	15.0428	15.2624	13.6140
15	1.8245	2.4727	1.1503	1.7258	1.3121	1.4154	1.2109

Table S3: Interaction energies, kJ/mol

	aDZ		aTZ		aQZ		DZ-F12		TZ-F12	
	β_{ref}	β_{opt}	β_{ref}	β_{opt}	β_{ref}	β_{opt}	β_{ref}	β_{opt}	β_{ref}	β_{opt}
$H_2O\ldots H_2O$	-4.906	-4.904	-4.868	-4.869	-4.822	-4.823	-4.799	-4.822	-4.802	-4.804
HCCH...HCCH	-1.395	-1.398	-1.386	-1.408	-1.366	-1.370	-1.345	-1.342	-1.367	-1.356
$CH_3OH\ldots CH_3OH$	-5.666	-5.664	-5.568	-5.577	-5.492	-5.497	-5.485	-5.515	-5.488	-5.492
$H_2O\ldots CH_3OH$	-5.544	-5.543	-5.471	-5.477	-5.413	-5.417	-5.388	-5.416	-5.402	-5.405
$HCCH\ldots H_2O$	-2.943	-2.943	-2.843	-2.854	-2.803	-2.803	-2.760	-2.771	-2.779	-2.771

Table S4: Ionization Potentials, kJ/mol

		$B_2 \rightarrow B_2^+$	$C_2 \rightarrow C_2^+$	$N_2 \rightarrow N_2^+$	$O_2 \rightarrow O_2^+$	$F_2 \rightarrow F_2^+$
DZ-F12	β_{ref}	8.705	12.362	15.691	11.146	16.032
DZ-F12	β_{opt}	8.706	12.364	15.695	11.158	16.047
TZ-F12	β_{ref}	8.727	12.380	15.735	11.220	16.102
TZ-F12	β_{opt}	8.730	12.382	15.740	11.227	16.106
QZ-F12	β_{ref}	8.734	12.386	15.750	11.247	16.131
QZ-F12	β_{opt}	8.737	12.388	15.753	11.250	16.134
5Z-F12	β_{ref}	8.735	12.387	15.755	11.255	16.139
5Z-F12	β_{opt}	8.738	12.389	15.757	11.257	16.140
aDZ	β_{ref}	8.695	12.352	15.638	11.044	15.940
aDZ	β_{opt}	8.695	12.352	15.638	11.045	15.942
aTZ	β_{ref}	8.731	12.383	15.732	11.212	16.095
aTZ	β_{opt}	8.730	12.381	15.733	11.215	16.097
aQZ	β_{ref}	8.736	12.388	15.751	11.247	16.130
aQZ	β_{opt}	8.736	12.386	15.751	11.247	16.130
a5Z	β_{ref}	8.736	12.388	15.756	11.255	16.139
a5Z	β_{opt}	8.737	12.388	15.757	11.256	16.140
CBS		8.738	12.389	15.760	11.260	16.144

Table S5: Atomization energies, kJ/mol

basis		HF	F_2	H_2O	N_2	NH_3	OH	CH
aDZ	β_{ref}	-175.19	-289.82	-299.55	-419.83	-373.26	-150.30	-103.32
aDZ	β_{opt}	-175.27	-290.00	-299.69	-420.19	-373.44	-150.38	-103.35
aTZ	β_{ref}	-176.03	-284.51	-303.44	-422.51	-382.29	-152.70	-107.79
aTZ	β_{opt}	-176.10	-285.04	-303.68	-423.31	-382.74	-152.90	-107.98
aQZ	β_{ref}	-177.90	-285.29	-306.16	-426.54	-385.73	-154.32	-108.96
aQZ	β_{opt}	-177.87	-285.25	-306.20	-426.70	-385.86	-154.36	-109.00
a5Z	β_{ref}	-177.31	-284.44	-305.73	-426.73	-385.57	-154.09	-108.95
a5Z	β_{opt}	-177.40	-284.54	-305.89	-427.06	-385.82	-154.19	-109.03
a6Z	β_{ref}	-177.23	-284.30	-305.64	-426.78	-385.54	-154.06	-108.94
a6Z	β_{opt}	-177.29	-284.37	-305.77	-427.08	-385.76	-154.13	-109.02
a7Z	β_{ref}	-177.23	-284.27	-305.68	-426.81	-385.62	-154.07	-108.97
a7Z	β_{opt}	-177.27	-284.31	-305.77	-427.06	-385.77	-154.13	-109.03
DZ-F12	β_{ref}	-172.19	-278.97	-296.43	-415.48	-372.76	-148.78	-104.21
DZ-F12	β_{opt}	-173.31	-281.81	-298.18	-418.67	-374.47	-149.67	-104.49
TZ-F12	β_{ref}	-175.56	-283.16	-302.46	-422.87	-381.32	-152.26	-107.68
TZ-F12	β_{opt}	-176.05	-284.18	-303.44	-424.40	-382.56	-152.79	-108.13
QZ-F12	β_{ref}	-176.73	-283.96	-304.80	-425.50	-384.27	-153.58	-108.52
QZ-F12	β_{opt}	-177.02	-284.31	-305.37	-426.42	-385.15	-153.88	-108.82
5Z-F12	β_{ref}	-176.99	-284.09	-305.29	-426.34	-385.03	-153.87	-108.73
5Z-F12	β_{opt}	-177.21	-284.29	-305.66	-426.92	-385.62	-154.07	-108.95
